# Ultrasensitive and rapid identification of ESRI developer- and piperacillin/tazobactam-resistant *Escherichia coli* by the MALDIpiptaz test

**DOI:** 10.1080/22221751.2022.2113746

**Published:** 2022-08-28

**Authors:** Angel Rodríguez Villodres, Lydia Gálvez Benítez, Manuel J. Arroyo, Gema Méndez, Luis Mancera, Andrea Vila Domínguez, José Antonio Lepe Jímenez, Younes Smani

**Affiliations:** a Clinical Unit of Infectious Diseases, Microbiology and Preventive Medicine, Virgen del Rocío University Hospital, Seville, Spain; b Institute of Biomedicine of Seville (IBiS), Virgen del Rocío University Hospital/CSIC/University of Seville, Seville, Spain; c Clover Bioanalytical Software, Av. del Conocimiento, 41, 18016 Granada, Spain; d Department of Molecular Biology and Biochemical Engineering, Andalusian Center of Developmental Biology, CSIC, University of Pablo de Olavide, Seville, Spain

**Keywords:** Piperacillin, tazobactam, *Escherichia coli*, resistance, beta-lactamase, beta-lactamase inhibitor, machine learning, algorithm

## Abstract

**Background:**

The excessive use of piperacillin/tazobactam (P/T) has promoted the emergence of P/T-resistant *Enterobacterales*. We reported that in *Escherichia coli*, P/T contributes to the development of extended-spectrum resistance to β-lactam/β-lactamase inhibitor (BL/BLI) (ESRI) in isolates that are P/T susceptible but have low-level resistance to BL/BLI. Currently, the detection of P/T resistance relying on conventional methods is time-consuming. To overcome this issue, we developed a cost-effective test based on MALDI-MS technology, called MALDIpiptaz, which aims to detect P/T resistance and ESRI developers in *E. coli*.

**Methods:**

We used automated Clover MS Data Analysis software to analyse the protein profile spectra obtained by MALDI-MS from a collection of 248 *E. coli* isolates (91 P/T-resistant, 81 ESRI developers and 76 P/T-susceptible). This software allowed to preprocess all the spectra to build different peak matrices that were analysed by machine learning algorithms.

**Results:**

We demonstrated that MALDIpiptaz can efficiently and rapidly (15 min) discriminate between P/T-resistant, ESRI developer and P/T-susceptible isolates and allowed the correct classification between ESRI developers from their isogenic resistance to P/T.

**Conclusion:**

The combination of excellent performance and cost-effectiveness are all desirable attributes, allowing the MALDIpiptaz test to be a useful tool for the rapid determination of P/T resistance in clinically relevant *E. coli* isolates.

## Introduction

Antimicrobial resistance is becoming one of the worst health crises of this century. The most alarming consequences is the spread of the resistance mechanisms that reduce the therapeutic usefulness of different antibiotic families, such as β-lactams. Over the past century, *Escherichia coli*, among other bacteria, has frequently exhibited resistance to β-lactams. In this context, β-lactamase inhibitors (BLIs) were developed to be used in combination with β-lactams to inhibit β-lactamase, thus allowing β-lactams to act unimpeded [1,2]. Among these combinations, piperacillin/tazobactam (P/T) is the most efficacious against *E. coli* and other gram-negative bacteria producing β-lactamases (mainly TEM enzymes) [1]. However, recent studies have reported that *E. coli* resistance to P/T is becoming increasingly prevalent worldwide [3–8].

Acquired resistance to P/T in *E. coli* arises from TEM-1 hyperproduction, evolution of inhibitor-resistant TEM variants and evolution of TEM variants with higher hydrolytic capacities [4,6–10]. Other β-lactamases, such as OXA-1 and AmpC enzymes, and porin loss are common causes of P/T resistance [11–15].

Notably, BL/BLI resistance in *E. coli* is a gradual and unidirectional process that extends from ampicillin/sulbactam (A/S) to P/T [4]. We previously characterized a new concept called extended-spectrum BL/BLI resistance (ESRI), which is defined as the acquisition of high-level resistance to BLs/BLIs (resistance to A/S, amoxicillin/clavulanic acid (A/C) and P/T) from low- (resistance to A/S and susceptibility to A/C and P/T) or moderate-level resistance to BLs/BLIs (resistance to A/S and A/C and susceptibility to P/T), in which *bla*_TEM_ plays an important role [4].

Currently, semiautomated systems (MicroScan, Vitek 2 and BD Phoenix) are well established worldwide to determine the susceptibility of P/T-generating results up to 18–20 h after bacterial identification [16]. In addition, molecular biology (e.g. conventional and real-time PCR or whole genome sequencing) is widely used for the detection of many antimicrobial resistance mechanisms [17,18]. Nonetheless, this may not be the best option for the detection of P/T resistance because these methods are not simple and are expensive [17].

Recently, a biochemical test, i.e. the ESRI test, was developed to detect P/T-resistant *E. coli* isolates and P/T-susceptible *E. coli* isolates but with capability for ESRI development (ESRI developers) [19]. This test is semirapid (within 3 h after bacterial identification by MALDI-MS) and easy to perform, but it is designed only for *E. coli* growing in haemoculture bottles rather than agar plates. However, fast detection of P/T-resistant *E. coli* isolates and ESRI developer isolates is one of the key issues in optimizing antimicrobial treatment, which would improve the clinical prognosis both in survival and in the absence of recurrence by resistant microorganisms.

To overcome these issues, we developed a cost-effective assay based on MALDI-MS technology, called MALDIpiptaz, which aims to detect P/T resistance and ESRI developers using a single bacterial colony in 15 min. Recent studies have reported that MALDI-MS is a rapid method for detecting colistin resistance in *E. coli*, *Klebsiella pneumoniae*, *Acinetobacter baumannii* and *Salmonella* enterica [20–23], methicillin resistance in *Staphylococcus aureus* [24], and carbapenemase-producing *Enterobacterales* [25–27].

Herein, we evaluated the potential of MALDI-MS for the detection of P/T resistance and ESRI development in *E. coli* isolates. We used automated Clover MS Data Analysis software (Clover BioSoft, Spain) to analyse the protein profile spectra obtained by MALDI-MS. This software allowed us to preprocess all the spectra to build different peak matrices that were analysed by machine learning algorithms. By using a large panel of *E. coli* isolates, we demonstrated that this test can efficiently discriminate between P/T-resistant, ESRI developer and P/T-susceptible isolates. This test also allowed the correct classification between ESRI developers from their isogenic resistance to P/T.

## Materials and methods

### Bacterial strains and culture conditions

We used a collection of non-duplicated 248 *E. coli* isolates, including 54 P/T-resistant and 81 ESRI developer isolates [19]. The 74 P/T-susceptible *E. coli* isolates were *wild-type*. The remaining 37 isolates were ESRI developers with already acquired resistance. These isolates were obtained from bloodstream and intraabdominal samples of patients with suspected bacteremia or intraabdominal infections at the Universities Hospitals of Virgen del Rocío of Seville, A Coruña and Son Espases of Mallorca between 2016 and 2021. Bacterial isolates were stored at −80 °C using cryopreservation vials (Deltalab, Barcelona, Spain). Bacterial isolates were seeded on a blood agar with a sterile loop before incubation in aerobic atmosphere at 37° for 18 h.

### Susceptibility testing

The P/T susceptibility profile was initially tested by broth microdilution using MicroScan Walk Away NM44 panels (Beckman Coulter, Inc., USA). MICs of P/T were subsequently confirmed using the standard broth microdilution method [28]. MICs were determined on original isolates and ESRI developers. The MIC susceptibility breakpoint of P/T in *E. coli* was determined according to the standard recommendations of EUCAST being susceptible ≤8 mg/L, resistant >8 mg/L and in the area of technical uncertainty (ATU) = 16 mg/L [28].

### MLST assay

Seven housekeeping genes (*adk*, *fumC*, *gyrB*, *icd*, *mdh*, *purA* and *recA*) were amplified by conventional PCR and analysed in the most of the studied isolates as we previously described [4].

### Detection and sequencing of *bla*_TEM_, *bla*_OXA−1_, and *bla*_SHV_ genes, and ESRI.

The *bla*_TEM_, *bla*_OXA−1_, and *bla*_SHV_ genes and ESRI were determined and analyzed in all studied isolates by PCR and ESRI test, respectively, as we previously described [19].

### MALDIpiptaz test

Bacterial strains were cultured on Columbia agar with 5% sheep blood. A single colony was smeared as a thin film directly onto a MALDI-TOF MS target (MBT Biotargets-96, Bruker Daltonik, Germany) as a homogeneous distribution of material. After the sample was dried, 1 µL of α-cyano-4-hydroxycinnamic acid matrix solution (Bruker Daltonik, Germany) was resuspended in the standard solution (acetonitrile 50%, water 47.5% and trifluoroacetic acid 2.5%) and overlayed in each sample position.

The bacterial solution and matrix were mixed directly on the target by pipetting, and the mix was air dried for at least 2 min. MALDI-TOF MS analysis was performed on a Bruker MicroFlex LT mass spectrometer (Bruker Daltonics Inc., GmbH, Germany). Spectra were acquired over a mass/charge (*m/z*) ratio ranging from 2,000 to 20,000. Each spot was measured using 120 laser shots at 60 Hz. FlexAnalysis and MBT Compass software (v4.1; Bruker Daltonics, Inc.) were used in the MS data analysis.

The external calibration was performed and following the Bruker’s recommendation by including the bacterial test standard (BTS; Bruker Daltonics, Germany) in each run before spectral acquisition.

### Peak analysis

For the classification of the P/T-susceptible and resistant isolates and ESRI developer isolates, their protein spectra were processed using Clover MS Data Analysis software (Clover Biosoft, Spain), as summarized in Table S1. The spectrum features from isolates were analysed by peak matrices built in the range of 2,000 m/z to 20,000 m/z. The spectra were first preprocessed to remove the noise by applying a Savitzky–Golay filter as smoothing and a top-hat filter for baseline subtraction. A spectral alignment with a replicate union process was then performed as follows: 1) shot spectra within the same spot were aligned to create an average spot spectrum per target position; 2) these average spectra were aligned with those from other spots of the same sample, and thus, one average spectrum per sample was obtained; and 3) average spectra from different isolates were then aligned together. Once the data were normalized in this step by total ion current (TIC) normalization, two different methods were tested for building the peak matrices. In the full spectrum method, peak matrices were built with all intensities of the entire spectra. On the other hand, using the threshold method, the peak matrices were built from all peaks with intensities higher than 1.0% of the maximum peak intensity (0.01 factor) of each spectrum.

For the classification of the isolates into selected categories, the peak matrices were then used as input data for three different machine learning supervised algorithms: PLS-DA, SVM and random forest. Stratified k-fold cross validation was used as internal validation, in which the dataset was randomly split into (k) balanced folds. From the folds obtained, k-1 were used for training the algorithm, and the remaining values were used as a blind set. This process was iterated k times, so each fold was tested in a blinded manner. Furthermore, this k-fold was repeated 20 times to obtain the average score and its relative error.

This classification assay was first applied to discriminate pairs of ESRI developers susceptible to P/T (original isolates) from those that were pressed *in vitro* to acquire P/T resistance (pressed isolates) [4], as a previous study to test the potential of the approach described above. The main assay was then divided into two steps as follows: 1) discrimination of the P/T-susceptible and ESRI developer isolates from P/T-resistant isolates and 2) discrimination of the P/T-susceptible isolates from ESRI developer isolates.

## Results

### Differentiation between phenotypic ESRI developers susceptible to P/T and their isogenic P/T-resistant *E. coli*

Three machine learning supervised algorithms, partial least squares discriminant analysis (PLS-DA), linear support vector machines (SVMs) and random forest (RF), were applied to the peak matrix generated by the full spectrum and threshold methods of 37 ESRI developers susceptible to P/T and their isogenic P/T-resistant isolates. The average results after 20 repetitions of the 10-fold assays are recorded in Table 1.

**Table 1.** Average results in accuracy terms by repeating 20 times a 10-fold cross validation analysis 20 times for all ML supervised algorithms for each method and step.

	PLS-DA		SVM		RF	
	Full Spectrum (%)	Threshold (%)	Full Spectrum (%)	Threshold (%)	Full Spectrum (%)	Threshold (%)
ESRI (original *vs*. pressed), N = 74	80.33 ± 0.68	78.51 ± 0.48	61.48 ± 0.22	57.43 ± 1.00	90.13 ± 0.22	87.50 ± 0.40
Step 1 (P/T-susceptible and ESRI (original)) *vs*. P/T-resistant, N = 209	92.85 ± 0.11	91.80 ± 0.10	77.14 ± 0.21	90.09 ± 0.04	96.92 ± 0.08	95.52 ± 0.14
Step 2 (P/T-susceptible *vs*. ESRI (original)) isolates, N = 157	74.33 ± 0.23	71.52 ± 0.29	61.56 ± 0.34	55.66 ± 0.27	87.77 ± 0.25	81.75 ± 0.29

PLS-DA: Partial Least Square Discriminant Analysis; SVM: Linear Support Vector Machines; RF: Random Forest.

Correct separation between both categories was achieved by applying the RF algorithm using the full spectrum and threshold methods, with 90.13% ± 0.22% and 87.50% ± 0.40% accuracy, respectively. In turn, PLS-DA and SVM achieved accuracies lower than 81% in both methods.

The 10-fold internal validation of the RF full spectrum method yielded an F1 score (the harmonic mean of the sensitivity and the accuracy of the model) of 91.43%, with a sensitivity and specificity of 86.49% and 97.3%, respectively. The positive and negative predictive values were 96.97% and 87.8%, respectively, assuming the isogenic P/T-resistant isolates as the positive category. Similar 10-fold scores, sensitivity and specificity percentages were obtained with the threshold method. Additionally, in the case of PLS-DA and SVM, the 10-fold precision was lower than 83%.

**Figure 1.**
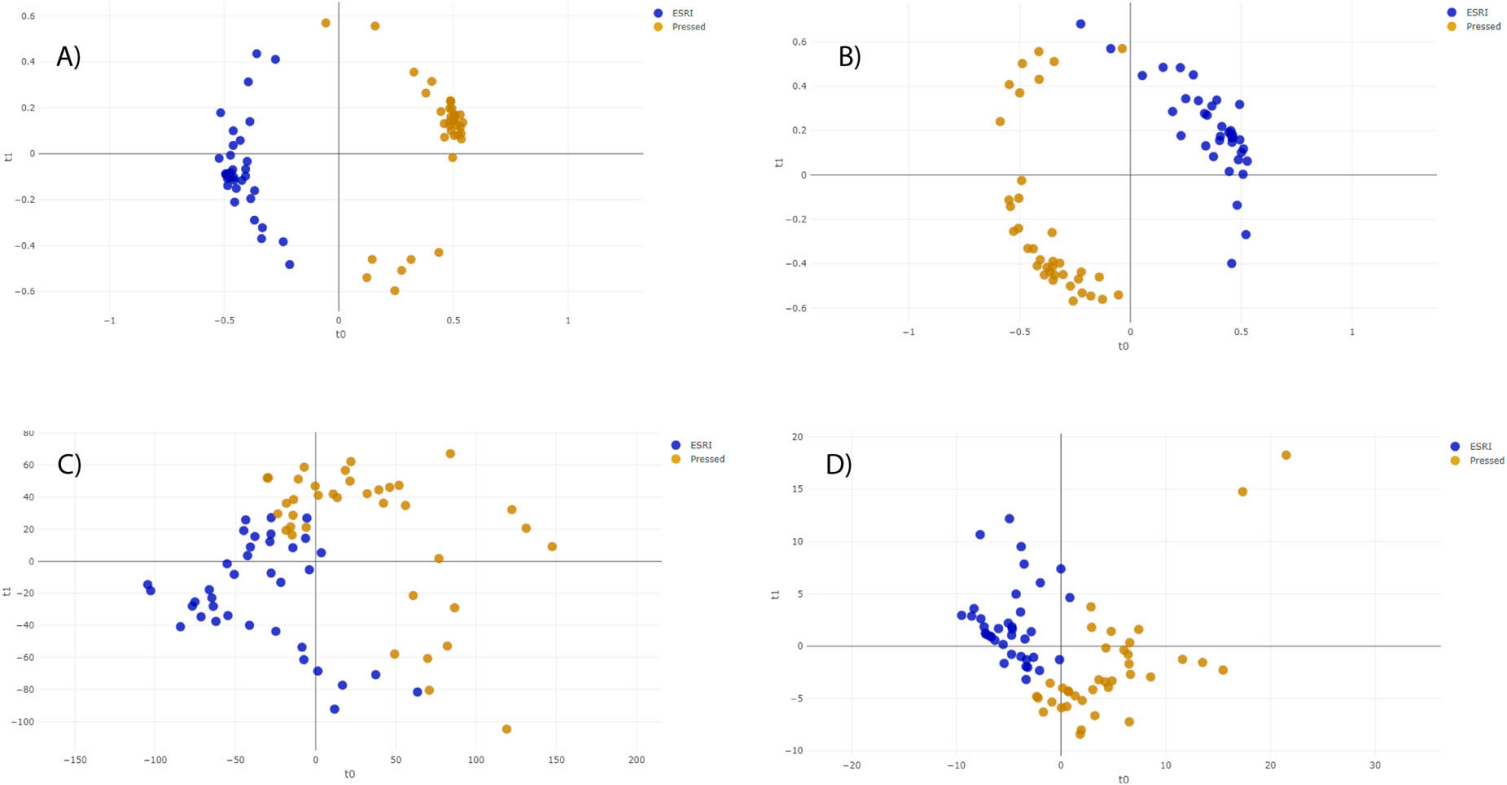
Distance plot of RF and PLS-DA for discrimination between ESRI developers susceptible to P/T and their isogenic isolates resistant to P/T. **A. C,** Full Spectrum method. **B. D**, Threshold method. ESRI: Extended spectrum resistance to BL/BLI susceptible to P/T; Pressed: ESRI isogenic isolates resistant to P/T after pressure with P/T; PLS-DA: partial least squares discriminant analysis; RF: random forest. t0 and t1 are two components of an algorithm called MDS that help to represent data from a distance matrix.

The distance plot of RF and PLS-DA analysis for discrimination between ESRI developers susceptible to P/T and their isogenic isolates resistant to P/T showed that the RF full spectrum and threshold methods achieved positive separation between both groups of isolates compared with the PLS-DA full spectrum and threshold methods (Figure 1). In the case of SVM full spectrum and threshold methods, distance plot analysis was not performed because principal component analysis (PCA) must be applied before, and it did not discriminate between samples (data not shown).

### Differentiation between phenotypic P/T-susceptible and P/T-resistant *E. coli*

The same three supervised algorithms were applied to the peak matrix generated by the full spectrum and threshold methods of 157 P/T-susceptible (53 of which were ESRI developers susceptible to P/T) and 53 P/T-resistant *E. coli*.

In the first step of the assay for the determination of P/T resistance, RF achieved 96.92% ± 0.08% and 95.52% ± 0.14% for the full spectrum and threshold methods, respectively (Table 1). In this step, the PLS-DA (in full spectrum and threshold methods) and SVM (in threshold method) algorithms also showed more than 90% accuracy (Table 1). The 10-fold internal validation of the RF full spectrum method yielded an F1 score of 94.23%, with a sensitivity and specificity of 92.45% and 98.73%, respectively. The positive and negative predictive values were 96.08% and 97.48%, respectively, assuming resistant isolates as the positive category. Similar results were achieved with PLS in both methods. Additionally, in the case of SVM, the 10-fold precision was 70% and 90.09% in the full spectrum and threshold methods, respectively.

**Figure 2.**
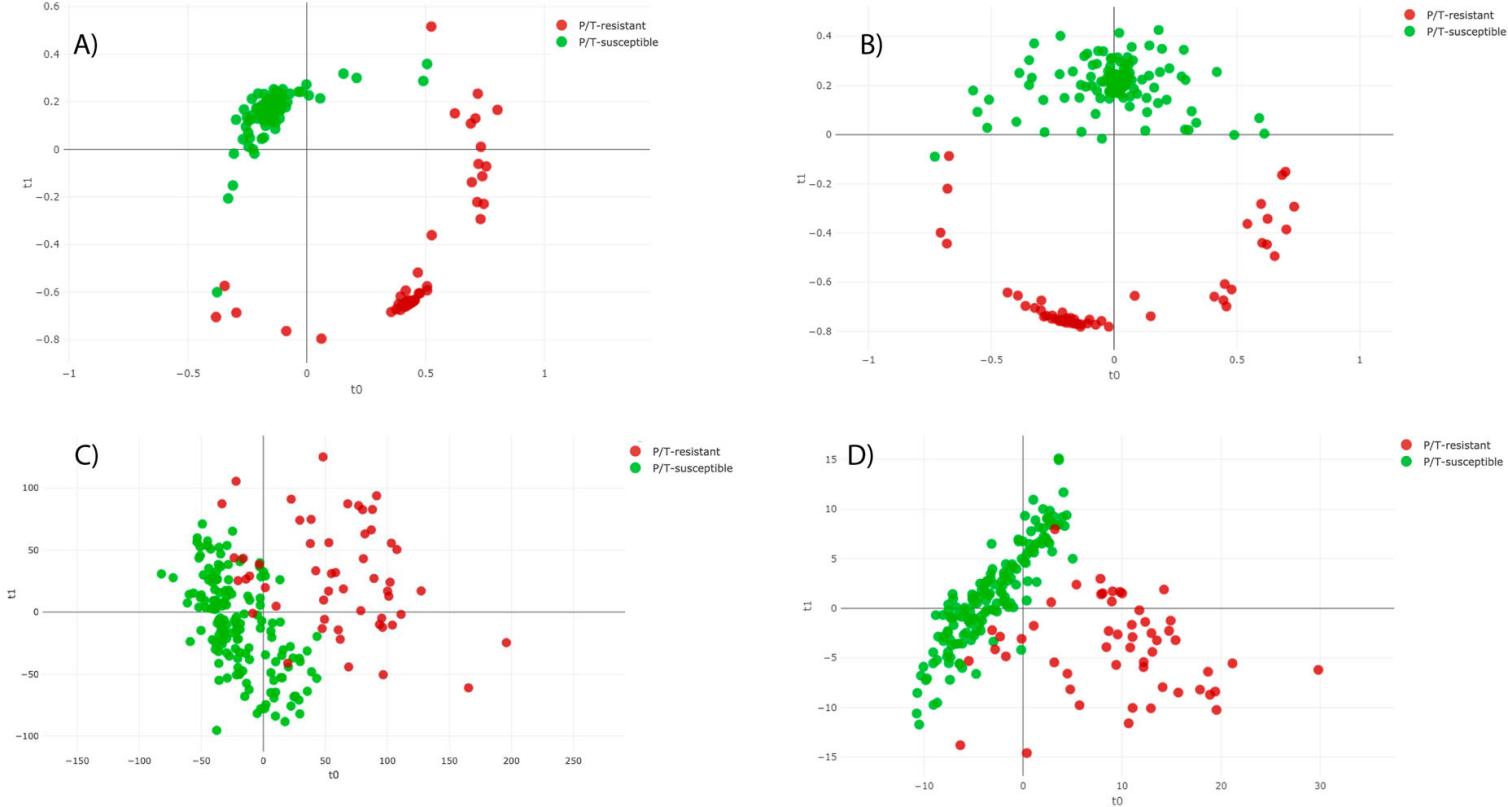
Distance plot of RF and PLS-DA for discrimination between P/T-susceptible and P/T-resistant *E. coli* (first step analysis). **A. C**, Full Spectrum method. **B. D**, Threshold method. PLS-DA: Partial Least Square Discriminant Analysis; RF: Random Forest. t0 and t1 are two components of an algorithm called MDS that help to represent data from a distance matrix.

The distance plot of RF and PLS-DA analysis for discrimination between P/T-susceptible and P/T-resistant *E. coli* showed that the RF full spectrum and threshold methods achieved positive separation between both groups of isolates compared with the PLS-DA full spectrum and threshold methods (Figure 2). In the case of SVM full spectrum and threshold methods, distance plot analysis was not performed because PCA did not discriminate between samples (data not shown).

### Differentiation between phenotypic P/T-susceptible and ESRI developer *E. coli*

To determine whether the MALDIpiptaz test is able to differentiate between P/T-susceptible and ESRI developer *E. coli*, we analysed two collections of P/T-susceptible *E. coli* (N = 76) and ESRI developers susceptible to P/T but with capability for ESRI development (N = 81).

RF analysis achieved the best accuracy for the classification of ESRI developers from susceptible isolates compared with PLS-DA and SVM analyses (Table 1). The RF algorithm achieved 87.77% ± 0.25% and 81.75% ± 0.29% accuracy for the full spectrum and threshold methods, respectively. In turn, PLS-DA and SVM in both methods showed accuracies lower than 75% and 62%, respectively.

The 10-fold internal validation of the RF full spectrum method yielded an F1 score of 86.79%, with 85.19% and 88.16% sensitivity and specificity, respectively, assuming ESRI developers were the positive category. The positive and negative predictive values were 88.46% and 84.81%, respectively. Additionally, in the case of PLS-DA and SVM, the 10-fold accuracy was lower than 75%.

**Figure 3.**
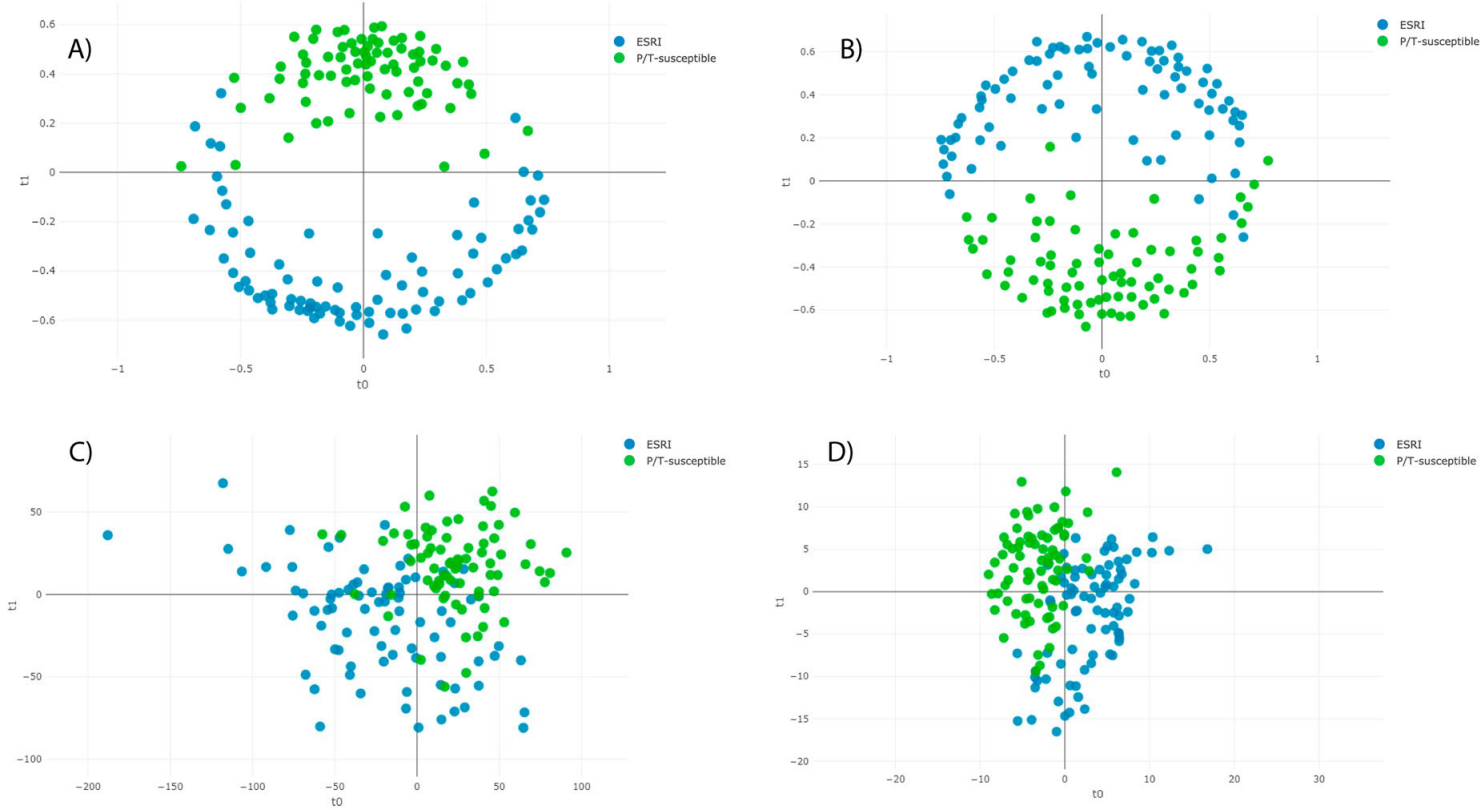
Distance plot of RF and PLS-DA for discrimination between P/T-susceptible and ESRI developer *Escherichia coli* (second step analysis). **A. C**, Full Spectrum method. **B. D**, Threshold method. PLS-DA: Partial Least Square Discriminant Analysis; RF: Random Forest. t0 and t1 are two components of an algorithm called MDS that help to represent data from a distance matrix.

The distance plot of RF and PLS-DA analysis for discrimination between P/T-susceptible and ESRI developer *E. coli* showed that the RF full spectrum and threshold methods achieved positive separation between both groups of isolates compared with the PLS-DA full spectrum and threshold methods (Figure 3). In the case of SVM full spectrum and threshold methods, distance plot analysis was not performed because PCA did not discriminate between samples (data not shown).

Differentiation between phenotypic P/T-susceptible and ESRI developer or P/T-resistant *E. coli* by peak feature importance study and ROC curves.

**Figure 4.**
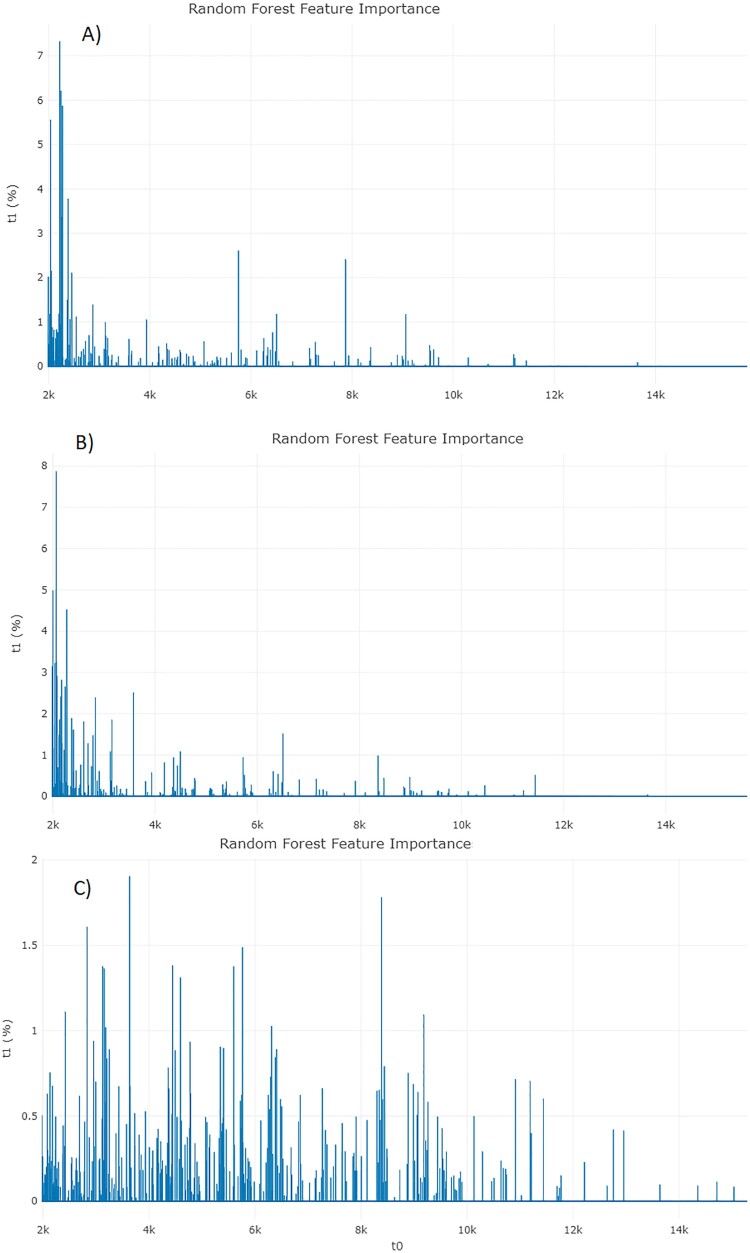
Feature importance from RF analysis from each assay. **A,** ESRI developers susceptible to P/T and their isogenic isolates resistant to P/T. **B,** P/T-susceptible and P/T-resistant *E. coli* (Step 1). **C,** P/T-susceptible and ESRI developers susceptible to P/T (Step 2). RF: Random Forest; ESRI: Extended Spectrum Resistance to BL/BLI susceptible to P/T.

Further discrimination in the three assays between (i) ESRI developer isolates susceptible to P/T and their isogenic isolates resistant to P/T, (ii) P/T-susceptible isolates and P/T-resistant isolates, and (iii) P/T-susceptible isolates and ESRI developer isolates susceptible to P/T was attempted using a different approach for peak analysis by the feature importance of RF using full spectrum method (Figure 4). In these features, the higher the feature importance is, the more important it is when splitting the samples in the classifier trees. The importance of a feature is computed as the (normalized, the values of the array sum to 100%) total reduction of the criterion brought by that feature. It is also known as the Gini importance. Feature importance was obtained from RF between phenotypic ESRI developers susceptible to P/T and their isogenic P/T-resistant isolate assays, showing a high area of interest from 2000 to 3500 m/z (Figure 4A). In addition, feature importance was also obtained from RF between phenotypic P/T-susceptible isolates and P/T-resistant isolate assays, showing a high area of interest from 2000 to 3500 m/z (Figure 4B). On the other hand, the distribution of the feature importance in differentiation between P/T-susceptible isolates and ESRI developer isolates susceptible to P/T was more homogeneous and did not show any characteristic range (Figure 4C), but instead the entire spectrum contributes equally to the classification.

**Figure 5.**
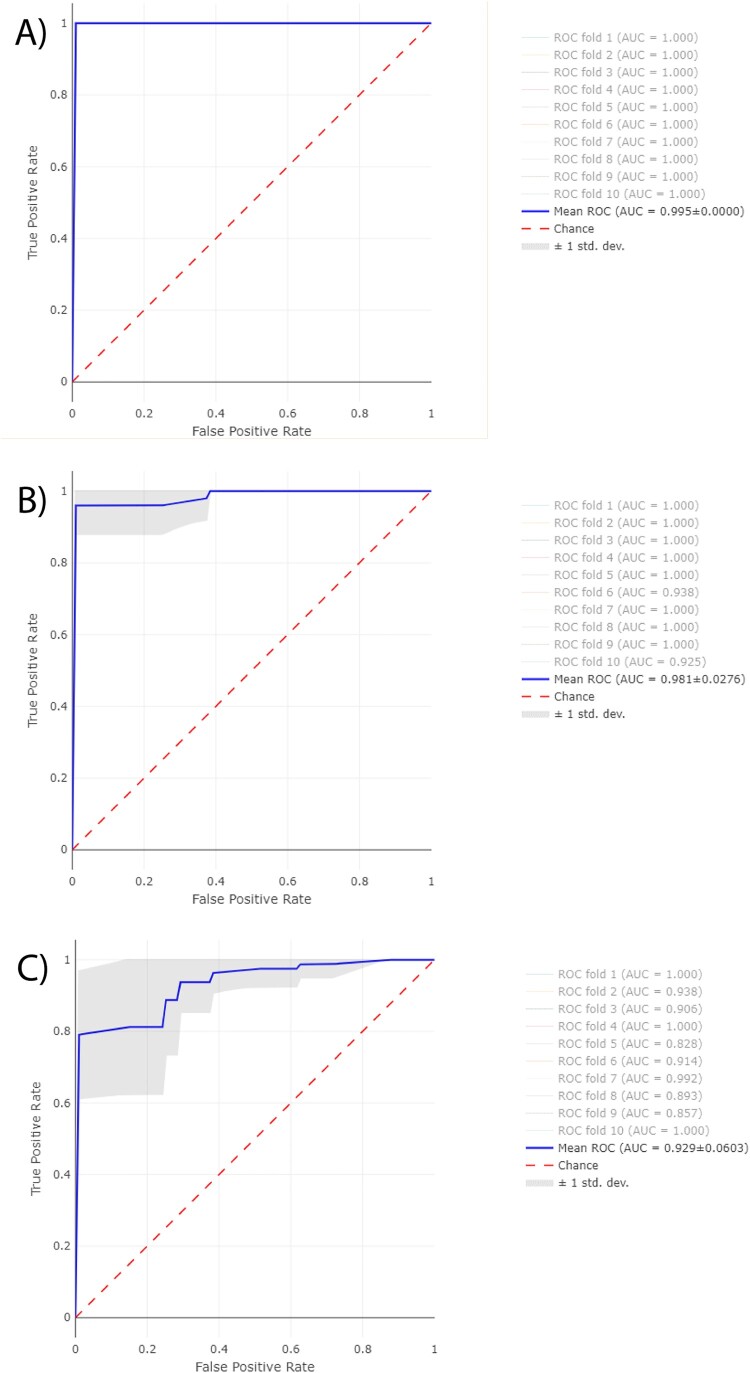
Average ROC curves from first 10-folds and their AUC values obtained from RF using Full Spectrum method to discriminate between **A,** ESRI developers susceptible to P/T and their isogenic isolates resistant to P/T **B,** P/T-susceptible and P/T-resistant *E. coli* and **C,** P/T-susceptible and ESRI developer *Escherichia coli.* Isogenic isolates resistant to P/T, resistant isolates to P/T and ESRI developer isolates susceptible to P/T were labelled as positive categories respectively.

Receiver Operating Characteristic (ROC) curves were obtained from RF for the same three assays described above (Figure 5). Furthermore, their area under the curve (AUC) were obtained for the first 10-folds of each assay. The average AUC-ROC was 0.995 ± 0.05 for ESRI developer isolates susceptible to P/T and their isogenic isolates resistant to P/T, 0.981 ± 0.02 for P/T-susceptible isolates and P/T-resistant isolates, and 0.929 ± 0.06 for P/T-susceptible isolates and ESRI developer isolates susceptible to P/T. Isogenic isolates resistant to P/T, the resistant isolates to P/T and ESRI developer isolates susceptible to P/T were labelled as positive categories for getting the previous results, respectively.

## Discussion

Owing to the production of β-lactamase, BL/BLI was developed for the treatment of severe bacterial infections. In *E. coli*, resistance to P/T results from activation of TEM and evolution of inhibitor-resistant TEM variants, among others [4,6–8,10], which have been related to greater 30-day mortality of patients with bloodstream infections by ceftriaxone-resistant *E. coli* and *Klebsiella* spp. in the MERINO clinical trial [11,29].

The conventional detection of P/T resistance remains time-consuming in clinical microbiology laboratories and relies solely on the determination of P/T MICs using a microdilution assay [28].

To provide a highly sensitive, rapid and cost-effective alternative method, we developed a diagnostic test based on MALDI-TOF MS and Clover Bioanalytical Software, called MALDIpiptaz, for the detection of ESRI developer- and P/T-resistant isolates of *E. coli*. After initial validation using a well-characterized collection of ESRI developer-, P/T-susceptible and P/T-resistant isolates [4,19], we performed a prospective evaluation on *E. coli* isolated from bloodstream and intraabdominal samples during which the Clover Bioanalytical Software and the MALDIpiptaz test precisely detected all ESRI developer- and P/T-resistant isolates.

Additionally, an important challenge for MALDIpiptaz is the detection of ESRI developers by *E. coli*. Therefore, differentiation between ESRI developer and P/T-susceptible isolates is important due to the higher potential for the development of P/T resistance by ESRI developers, which consequently might cause the failure of this antibiotic therapeutic regimen. In the future, this distinction may be crucial to use adequate initial and early antibiotic treatment for severe infections by *E. coli*.

Currently, this differentiation between both cell populations is only determined by molecular methods (real-time PCR and WGS) that have detected regulation in *bla*_TEM_ genes [4,6,8]. Of note, the ESRI developer’s isolates harboured one or more different beta-lactamase genes such as TEM, SHV and OXA-1 [19]. These molecular methods are costly, time-consuming and not easy to perform in clinical microbiology laboratories. The MALDIpiptaz test directly identifies ESRI developer isolates and differentiates them from P/T-susceptible and P/T-resistant isolates. More specifically, our test identifies a specific signature (series of peaks between 2000 and 3500 m/z) associated with ESRI developer- and P/T-resistant isolates, leading to the precise differentiation between ESRI developer-, P/T-resistant and susceptible isolates.

**Figure 6.**
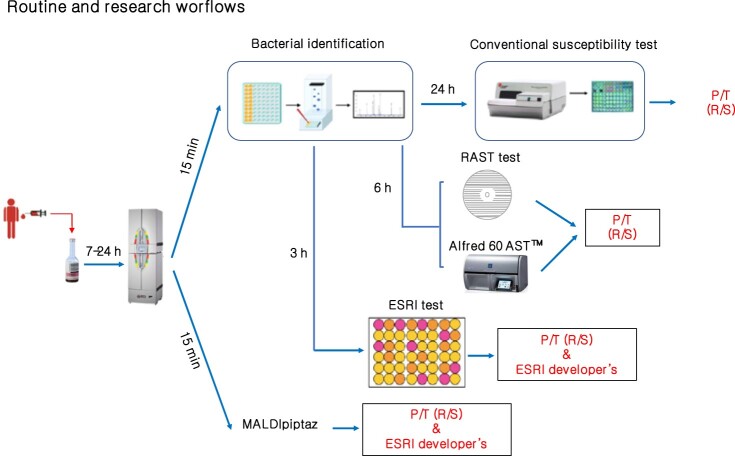
Routine and research workflows for rapid determination piperacillin/tazobactam resistance and ESRI developers by *E. coli*. P/T: piperacillin/tazobactam; R: Resistance; S: susceptible.

Recently, a simple semirapid colorimetric method, called the ESRI test, has been described for screening for ESRI developer- and P/T-resistant isolates of *E. coli* [19]. This method is based on β-lactam ring hydrolysis by β-lactamases. Although this method is cheap and easy to perform in a clinical microbiology laboratory, it is only designed for *E. coli* growing in blood culture bottles and requires an additional delay of at least 3 h compared with the MALDIpiptaz test (Figure 6). Similarly, automated commercial laser scattering based *in vitro* (Alfred 60 AST™) has provided P/T susceptibility in *E. coli* directly from positive blood culture bottles within 4–6 h [30]. In addition, RAST (rapid antimicrobial susceptibility testing) based on disk diffusion was established by EUCAST to determine within 6 h the susceptibility of P/T in *E. coli* from positive blood culture [31,32].

Interestingly, the MALDIpiptaz test could easily be incorporated into routine workflows because a MALDI-TOF MS-based approach for bacterial identification from isolate colonies within 15 min is now carried out routinely in many clinical microbiology laboratories [33], and a large collection of bacterial isolates can be analysed on the same MALDI-TOF target (between 96 positions are available on standard reusable MALDI target plates) and tested in the same run. However, we need to note that routine use of the MALDIpiptaz test will require online access on the Clover Bioanalytical website. The price per sample for rapid detection of antimicrobial resistance using MALDI-TOF MS technologies is usually from 1 to 10$ [34]. MALDIpiptaz test and software prices will be in that range per sample and should not add a significant amount to that.

The k-fold method used in this work allows researchers to exploit all dataset samples as blind and training sets when the number of samples is small. Furthermore, this method has been used in large datasets too [35], proving the usefulness of this method even when more formal validation is performed. That way, we have been able to achieve good results with no more than 1% of variability between k-fold tests by stratifying the folds of the method, considering the categories of the samples to always obtain a balanced fold. Nevertheless, further studies of this models, by including a validation set, will improve the models.

Finally, the MALDIpiptaz test is (i) rapid (15 min), while the confirmation of P/T resistance using conventional methods would have required an additional 24–48 h, and (ii) a precise diagnostic tool, which represents a major advance in the detection of ESRI developer- and P/T-resistant isolates of *E. coli*.

## Conclusions

We identified the MALDIpiptaz test using Clover MS Data Analysis software as a new diagnostic tool for the determination of ESRI developer- and P/T-resistant *E. coli*. The combination of excellent performance and cost-effectiveness are all desirable attributes that will allow further commercialization of the MALDIpiptaz test to use adequate antibiotic treatment for severe infections by *E. coli*.

## Supplementary Material

Supplemental Material

## Data Availability

The data that support the findings of this study are available from the corresponding author upon reasonable request.
