# Supplementary material for: Ultrasensitive and rapid identification of ESRI developer- and piperacillin/tazobactam-resistant Escherichia coli by the MALDIpiptaz test
Source: Emerg Microbes Infect. 2022 Aug 28;11(1):2034–44. doi: 10.1080/22221751.2022.2113746 (PMC9423838; doi:10.1080/22221751.2022.2113746)
Supplement: Supplemental Material [file TEMI_A_2113746_SM2921.docx]

**Table S1.** Parameters applied for peak matrices built in each assay and method.

| **Process** | **Parameters applied** |
| --- | --- |
| Baseline Substraction | Tophat filter  Factor: 0.02 |
| Smoothing | Sviztky-Golay filter   - Window length: 11 - Polynomial order: 3 |
| Alignment | Allowed shift: Medium  Mass tolerance for aligning the spectra:   - Constant tolerance: 3 Da - Linear Mass tolerance: 900 ppm |
| Normalization | - Total Ion Count (TIC) |
| Peaks | a) Full Spectrum:   - Skip this step   b) By threshold:   - Peak threshold: 0.01 Da |
| Merge | a) Full Spectrum method:   - Constant tolerance: 0.5 Da   b) Threshold method:   - Constant tolerance: 1 Da - Linear Mass tolerance: 300 ppm |

**Table S2. Clinical isolates of *Escherichia coli* susceptible to P/T.**

| **Isolate (*n* = 76)** | **Origin** | **ST** | **CC** | **Resistance mechanism** |
| --- | --- | --- | --- | --- |
| C1-74 | Blood | 101 | 101 | NEG |
| C1-83 | Blood | 12 | 12 | NEG |
| C1-90 | Blood | 359 | UK | NEG |
| C1-95 | Blood | 428 | 428 | NEG |
| C1-97 | Blood | 69 | 69 | NEG |
| C1-100 | Blood | UK | UK | NEG |
| C1-110 | Blood | 697 | 10 | NEG |
| C1-111 | Blood | 10 | 10 | NEG |
| C1-128 | Blood | 12 | 12 | NEG |
| C1-138 | Blood | ST345 | UK | NEG |
| C1-144 | Blood | ST315 | 38 | NEG |
| C1-146 | Blood | ST131 | 131 | NEG |
| C1-152 | Blood | 533 | UK | NEG |
| C1-155 | Blood | 101 | 101 | NEG |
| C1-157 | Blood | 13 | 13 | NEG |
| C1-168 | Blood | 74 | 73 | NEG |
| C1-169 | Blood | ND | ND | NEG |
| C1-172 | Blood | 569 | UK | NEG |
| C1-177 | Blood | 372 | 372 | NEG |
| C2-4 | Bile | 58 | 155 | NEG |
| C2-8 | Bile | 3640 | UK | NEG |
| C2-12 | Bile | 12 | 12 | NEG |
| AEC-2 | Peritoneal fluid | 95 | 95 | NEG |
| AEC-3 | Bile | 131 | 131 | NEG |
| AEC-4 | Bile | 58 | 155 | NEG |
| AEC-5 | Intra-abdominal abscess | 429 | UK | NEG |
| AEC-6 | Bile | UK | UK | NEG |
| AEC-7 | Peritoneal fluid | ND | ND | NEG |
| AEC-8 | Peritoneal fluid | 3640 | UK | NEG |
| AEC-10 | Bile | UK | UK | NEG |
| AEC-11 | Peritoneal fluid | 53 | 53 | NEG |
| AEC-12 | Peritoneal fluid | 73 | 73 | NEG |
| AEC-14 | Intra-abdominal abscess | 36 | 5 | NEG |
| AEC-15 | Peritoneal fluid | 10 | 10 | NEG |
| AEC-16 | Peritoneal fluid | 10 | 10 | NEG |
| AEC-17 | Pus | ND | ND | NEG |
| AEC-18 | Peritoneal fluid | 648 | 648 | NEG |
| AEC-19 | Intra-abdominal abscess | 131 | 131 | NEG |
| AEC-20 | Intra-abdominal abscess | 567 | UK | NEG |
| AEC-22 | Peritoneal fluid | 44 | 10 | NEG |
| AEC-23 | Peritoneal fluid | 131 | 131 | NEG |
| AEC-25 | Intra-abdominal abscess | 73 | 73 | NEG |
| AEC-26 | Peritoneal fluid | 825 | UK | NEG |
| AEC-27 | Peritoneal fluid | 705 | 131 | NEG |
| AEC-29 | Intra-abdominal abscess | 131 | 131 | NEG |
| AEC-30 | Intra-abdominal abscess | UK | UK | NEG |
| AEC-31 | Bile | 74 | 73 | NEG |
| AEC-33 | Bile | 1982 | 131 | NEG |
| AEC-34 | Peritoneal fluid | 475 | 10 | NEG |
| AEC-35 | Peritoneal fluid | 14 | 14 | NEG |
| AEC-36 | Liver abscess | 14 | 14 | NEG |
| AEC-37 | Intra-abdominal abscess | 493 | 12 | NEG |
| AEC-39 | Intra-abdominal abscess | 12 | 12 | NEG |
| AEC-40 | Peritoneal fluid | 372 | 372 | NEG |
| AEC-41 | Peritoneal fluid | UK | UK | NEG |
| AEC-42 | Peritoneal fluid | 14 | 14 | NEG |
| AEC-43 | Peritoneal fluid | 372 | 372 | NEG |
| AEC-44 | Peritoneal fluid | 4508 | UK | NEG |
| AEC-45 | Intra-abdominal abscess | ND | ND | NEG |
| AEC-46 | Peritoneal fluid | UK | UK | NEG |
| AEC-47 | Peritoneal fluid | 569 | UK | NEG |
| AEC-49 | Intra-abdominal abscess | 69 | 69 | NEG |
| AEC-50 | Peritoneal fluid | 131 | 131 | NEG |
| AEC-52 | Peritoneal fluid | ND | ND | NEG |
| AEC-54 | Intra-abdominal abscess | 69 | 69 | NEG |
| AEC-55 | Peritoneal fluid | ND | ND | NEG |
| AEC-56 | Peritoneal fluid | 14 | 14 | NEG |
| AEC-57 | Peritoneal fluid | 131 | 131 | NEG |
| AEC-58 | Peritoneal fluid | 307 | 93 | NEG |
| AEC-59 | Intra-abdominal abscess | ND | ND | NEG |
| AEC-60 | Intra-abdominal abscess | 542 | UK | NEG |
| AEC-61 | Peritoneal fluid | UK | UK | NEG |
| AEC-62 | Peritoneal fluid | ND | ND | NEG |
| AEC-63 | Peritoneal fluid | ND | ND | NEG |
| AEC-64 | Intra-abdominal abscess | 141 | UK | NEG |
| AEC-66 | Peritoneal fluid | 93 | 10 | TEM-1 |

ST: sequence type, CC: clonal complex, UK: unknown ST, ND: not determined.

NEG: negative result in relation to studied resistance mechanisms (beta-lactamases).

**Table S3. Clinical isolates of *Escherichia coli* that were ESRI developers and had positive results in the ESRI test.**

| **Isolate (*n* = 81)** | **Origin** | **ST** | **CC** | **Resistance mechanism** |
| --- | --- | --- | --- | --- |
| C1-8 | Blood | 3640 | UK | NEG |
| C1-31 | Blood | 95 | 95 | TEM-40 |
| C1-38 | Blood | 12 | 12 | TEM-35 |
| C1-48 | Blood | 58 | 155 | TEM-1 |
| C1-65 | Blood | 744 | 10 | TEM-1 |
| C1-72 | Blood | 533 | UK | TEM-1 |
| C1-81 | Blood | 131 | 131 | TEM-30 |
| C1-91 | Blood | 197 | UK | NEG |
| C1-93 | Blood | 428 | 428 | SHV |
| C1-102 | Blood | 95 | 95 | TEM-1 |
| C1-103 | Blood | 131 | 131 | TEM-1 |
| C1-106 | Blood | 131 | 131 | TEM-1 |
| C1-108 | Blood | 648 | UK | TEM-1 |
| C1-118 | Blood | 131 | 131 | OXA-1 |
| C1-120 | Blood | 73 | 73 | TEM-1 |
| C1-121 | Blood | 80 | 568 | TEM-1/SHV |
| C1-126 | Blood | 12 | 12 | TEM-1 |
| C1-129 | Blood | 131 | 131 | TEM-1 |
| C1-130 | Blood | 10 | 10 | TEM-1 |
| C1-141 | Blood | 131 | 131 | TEM-1/SHV |
| C1-142 | Blood | 58 | 155 | TEM-135/SHV |
| C1-153 | Blood | 533 | UK | TEM-1/SHV |
| C1-159 | Blood | 95 | 95 | TEM-1 |
| C1-160 | Blood | UK | UK | TEM-1 |
| C1-162 | Blood | ND | ND | NEG |
| C1-164 | Blood | 10 | 10 | TEM-1 |
| C1-171 | Blood | 3948 | 131 | NEG |
| C1-174 | Blood | ND | ND | TEM-1 |
| C1-175 | Blood | ND | ND | TEM-1 |
| C1-183 | Blood | 648 | UK | TEM-1 |
| C1-187 | Blood | 10 | 10 | TEM-1 |
| C1-190 | Blood | 65 | UK | SHV |
| C1-191 | Blood | 372 | 372 | TEM-1 |
| C1-306 | Blood | 746 | 10 | TEM-1 |
| C2-47 | Intra-abdominal abscess | 569 | UK | TEM-1 |
| C2-49 | Peritoneal fluid | 69 | 69 | TEM-1 |
| C2-54 | Intra-abdominal abscess | 69 | 69 | TEM-1 |
| PT3 | Blood | 88 | 23 | TEM-1 |
| ES-1 | Blood | ND | ND | TEM-1 |
| ES-2 | Blood | ND | ND | TEM-1 |
| ES-3 | Blood | ND | ND | TEM-1 |
| ES-4 | Blood | ND | ND | TEM-1 |
| ES-5 | Blood | ND | ND | TEM-1 |
| ES-6 | Blood | ND | ND | TEM-1 |
| ES-7 | Blood | ND | ND | TEM-1 |
| ES-8 | Blood | ND | ND | TEM-1 |
| ES-9 | Blood | ND | ND | TEM-1 |
| ES-10 | Blood | ND | ND | TEM-1 |
| ES-11 | Blood | ND | ND | TEM-1 |
| ES-12 | Blood | ND | ND | TEM-1 |
| ES-13 | Blood | ND | ND | TEM-1 |
| ES-14 | Blood | ND | ND | TEM-1 |
| ES-15 | Blood | ND | ND | TEM-1 |
| ES-16 | Blood | ND | ND | TEM-1 |
| ES-17 | Blood | ND | ND | TEM-1 |
| ES-18 | Blood | ND | ND | NEG |
| ES-28 | Blood | ND | ND | TEM-1 |
| ES-29 | Blood | ND | ND | TEM-1 |
| ES-30 | Blood | ND | ND | TEM-1 |
| ES-31 | Blood | ND | ND | TEM-1 |
| ES-32 | Blood | ND | ND | TEM-1 |
| ES-34 | Blood | ND | ND | TEM-1 |
| ES-35 | Blood | ND | ND | TEM-1 |
| ES-36 | Blood | ND | ND | TEM-1 |
| ES-37 | Blood | ND | ND | TEM-1 |
| ES-38 | Blood | ND | ND | TEM-1 |
| ES-39 | Blood | ND | ND | TEM-1 |
| ES-41 | Blood | ND | ND | TEM-1 |
| ES-42 | Blood | ND | ND | TEM-1 |
| ES-43 | Blood | ND | ND | TEM-1 |
| ES-44 | Blood | ND | ND | TEM-1 |
| ES-45 | Blood | ND | ND | TEM-1 |
| ES-47 | Blood | ND | ND | TEM-1 |
| ES-48 | Blood | ND | ND | TEM-1 |
| ES-49 | Blood | ND | ND | TEM-1 |
| ES-50 | Blood | ND | ND | TEM-1 |
| ES-51 | Blood | ND | ND | TEM-1 |
| ES-52 | Blood | ND | ND | TEM-1 |
| ES-53 | Blood | ND | ND | TEM-1 |
| ES-54 | Blood | ND | ND | TEM-1 |
| ES-55 | Blood | ND | ND | TEM-1 |

ST: sequence type, CC: clonal complex, UK: unknown ST, ND: not determined.

NEG: negative result in relation to studied resistance mechanisms (beta-lactamases).

**Table S4. Clinical isolates of Escherichia coli that were ESRI developers with already acquired resistance to P/T.**

| **Isolate PRESSED (*n* = 37)** | **Origin** | **ST** | **CC** | **Resistance mechanism** |
| --- | --- | --- | --- | --- |
| C1-8 | Blood | 3640 | UK | NEG |
| C1-31 | Blood | 95 | 95 | TEM-40 |
| C1-38 | Blood | 12 | 12 | TEM-35 |
| C1-48 | Blood | 58 | 155 | TEM-1 |
| C1-65 | Blood | 744 | 10 | TEM-1 |
| C1-72 | Blood | 533 | UK | TEM-1 |
| C1-81 | Blood | 131 | 131 | TEM-30 |
| C1-91 | Blood | 197 | UK | NEG |
| C1-93 | Blood | 428 | 428 | SHV |
| C1-102 | Blood | 95 | 95 | TEM-1 |
| C1-103 | Blood | ND | ND | TEM-1 |
| C1-106 | Blood | 131 | 131 | TEM-1 |
| C1-108 | Blood | 648 | UK | TEM-1 |
| C1-118 | Blood | 131 | 131 | OXA-1 |
| C1-120 | Blood | 73 | 73 | TEM-1 |
| C1-121 | Blood | 80 | 568 | TEM-1/SHV |
| C1-126 | Blood | 12 | 12 | TEM-1 |
| C1-129 | Blood | 131 | 131 | TEM-1 |
| C1-130 | Blood | 10 | 10 | TEM-1 |
| C1-141 | Blood | 131 | 131 | TEM-1/SHV |
| C1-142 | Blood | 58 | 155 | TEM-135/SHV |
| C1-153 | Blood | 533 | UK | TEM-1/SHV |
| C1-159 | Blood | 95 | 95 | TEM-1 |
| C1-160 | Blood | UK | UK | TEM-1 |
| C1-162 | Blood | ND | ND | NEG |
| C1-164 | Blood | 10 | 10 | TEM-1 |
| C1-171 | Blood | ND | ND | NEG |
| C1-174 | Blood | ND | ND | TEM-1 |
| C1-175 | Blood | ND | ND | TEM-1 |
| C1-183 | Blood | 648 | UK | TEM-1 |
| C1-187 | Blood | ND | ND | TEM-1 |
| C1-190 | Blood | ND | ND | SHV |
| C1-191 | Blood | 372 | 372 | TEM-1 |
| C1-306 | Blood | 746 | 10 | TEM-1 |
| C2-47 | Intra-abdominal abscess | 569 | UK | TEM-1 |
| C2-49 | Peritoneal fluid | 69 | 69 | TEM-1 |
| C2-54 | Intra-abdominal abscess | 69 | 69 | TEM-1 |

ST: sequence type, CC: clonal complex, UK: unknown ST, ND: not determined.

NEG: negative result in relation to studied resistance mechanisms (beta-lactamases).

**Table S5. Clinical isolates of *Escherichia coli* that were resistant to P/T.**

| **Isolate (*n* = 54)** | **Origin** | **ST** | **CC** | **Resistance mechanism** |
| --- | --- | --- | --- | --- |
| C1-1 | Blood | 2230 | 23 | TEM-1 |
| C1-23 | Blood | 131 | 131 | TEM-1 |
| C1-82 | Blood | 95 | 95 | TEM-1 |
| C1-94 | Blood | 428 | 428 | TEM-1 |
| C1-109 | Blood | 197 | UK | SHV |
| C1-116 | Blood | 131 | 131 | TEM-1 |
| C1-136 | Blood | 10 | 10 | TEM-1/SHV |
| C1-166 | Blood | UK | UK | TEM-1 |
| C1-189 | Blood | 648 | UK | TEM-1 |
| C1-239 | Blood | 131 | 131 | TEM-1 |
| C1-436 | Blood | UK | UK | TEM-1 |
| C2-23 | Bile | 131 | 131 | TEM-1 |
| C2-45 | Peritoneal fluid | 117 | UK | TEM-1 |
| C2-48 | Intra-abdominal abscess | 69 | 69 | TEM-1 |
| C2-57 | Bile | 131 | 131 | NEG |
| C2-72 | Intra-abdominal abscess | 569 | UK | TEM-1 |
| C2-74 | Peritoneal fluid | 569 | UK | TEM-1 |
| C2-82 | Hepatic abscess | 155 | 155 | TEM-1/SHV |
| C2-95 | Peritoneal fluid | 197 | UK | TEM-1 |
| C2-103 | Peritoneal fluid | 2230 | 23 | TEM-1 |
| C2-106 | Peritoneal fluid | 1485 | 648 | OXA-1 |
| C2-113 | Intra-abdominal abscess | 80 | 568 | TEM-1/SHV |
| C2-116 | Intra-abdominal abscess | 131 | 131 | TEM-1 |
| C2-136 | Peritoneal fluid | 10 | 10 | TEM-84 |
| C2-146 | Peritoneal fluid | UK | UK | TEM-1 |
| C2-147 | Intra-abdominal abscess | 569 | UK | TEM-1 |
| PT4 | Blood | 88 | 23 | TEM-1 |
| PTR1 | Blood | 131 | 131 | OXA-1 |
| PTR2 | Blood | 44 | 10 | TEM-1 |
| PTR3 | Blood | 88 | 23 | NEG |
| PTR4 | Blood | 57 | 350 | NEG |
| PTR5 | Blood | 2522 | UK | TEM-1 |
| PTR7 | Blood | 361 | UK | NEG |
| PTR8 | Blood | ND | ND | OXA-1 |
| PTR9 | Blood | ND | ND | TEM-1/OXA-1 |
| PTR11 | Blood | UK | UK | TEM-1 |
| PTR12 | Blood | UK | UK | NEG |
| PTR14 | Blood | ND | ND | NEG |
| PTR15 | Blood | ND | ND | OXA-1 |
| PTR16 | Blood | UK | UK | TEM-1 |
| PTR17 | Blood | UK | UK | TEM-1/OXA-1 |
| PTR19 | Blood | ND | ND | OXA-1 |
| PTR20 | Blood | ND | ND | TEM-1 |
| AEC-1 | Bile | 95 | 95 | TEM-1 |
| AEC-9 | Peritoneal fluid | 10 | 10 | TEM-1 |
| AEC-13 | Bile | 538 | 538 | NEG |
| AEC-21 | Intra-abdominal abscess | 92 | 127 | TEM-1 |
| AEC-24 | Peritoneal fluid | 10 | 10 | TEM-1 |
| AEC-32 | Liver abscess | 320 | 69 | TEM-1 |
| AEC-38 | Intra-abdominal abscess | 93 | 10 | TEM-1 |
| AEC-48 | Peritoneal fluid | 162 | 13 | TEM-1 |
| AEC-51 | Peritoneal fluid | 53 | 53 | TEM-1 |
| AEC-53 | Peritoneal fluid | 80 | 568 | SHV |
| AEC-69 | Pus | 93 | 10 | TEM-1 |

ST: sequence type, CC: clonal complex, UK: unknown ST, ND: not determined.

NEG: negative result in relation to studied resistance mechanisms (beta-lactamases).
